# Extracapillary immunoglobulin A nephropathy glomerulonephritis discovered during the course of pulmonary tuberculosis: a case report

**DOI:** 10.11604/pamj.2026.53.31.45089

**Published:** 2026-01-26

**Authors:** Asma Bettaieb, Raja Aoudia, Meriam Khadhar, Sarra Hadded, Hanene Gaied, Ines Moussa, Leila Douik El Gharbi, Mouna Jerbi, Rym Goucha

**Affiliations:** 1 Department of Nephrology, Mongi Slim Hospital, Tunis, Tunisia,; 2 Laboratory of Kidney Disease (LR00SP01), Charles Nicolle Hospital, Tunis, Tunisia,; 3 Faculty of Medicine, University of Tunis El Manar, Tunis, Tunisia,; 4 Pneumology Department D, Abderrahmen Mami Hospital, Ariana, Tunisia

**Keywords:** immunoglobulin A nephropathy, tuberculosis, renal failure, crescentic glomerulonephritis, case report

## Abstract

Few cases of immunoglobulin A nephropathy (IgAN) associated with tuberculosis have been described in the literature. We report an atypical case of extracapillary IgA glomerulonephritis discovered during the course of pulmonary tuberculosis. The patient, a 45-year-old diagnosed with pulmonary tuberculosis, presented with mixed nephrotic syndrome, characterized by hypertension, hematuria, and acute renal failure. We completed a renal biopsy which showed IgAN with diffuse crescents, classified as (M1E1S1T0C2) according to the Oxford classification. The patient was treated with high-dose of intravenous corticosteroids followed by oral tapering. The antituberculous quadruple therapy was extended to 2.5 months then followed by dual therapy. The course was marked by recovery from pulmonary tuberculosis and a significant improvement in creatinine levels, from 504 µmol/l to 262 µmol/L in five months, but the nephrotic syndrome persisted. In the absence of specific guidelines, this case demonstrates that individualized corticosteroid therapy may be considered in extracapillary IgA glomerulonephritis associated with tuberculosis.

## Introduction

Immunoglobulin A nephropathy (IgAN) is the most common primary glomerulonephritis worldwide [1]. Its clinical presentation is highly polymorphic, sometimes with atypical modes of onset explaining a wide range of histological changes described [2]. Infections, particularly of the respiratory tract, may play a triggering role in the pathogenesis of IgAN [3,4]. In fact, infections can cause abnormal mucosal immune responses leading to the production of galactose-deficient IgA, which then deposits with immune complexes in the kidneys´ glomerular mesangium and elicits an inflammatory reaction with tissue damage [3,4]. Tuberculosis has been linked to a wide range of glomerular presentations such as amyloidosis and membranous nephropathy [4]. Few cases of IgAN associated with tuberculosis have been described in the literature. We report an atypical case of extracapillary IgA glomerulonephritis discovered during the course of pulmonary tuberculosis.

## Patient and observation

**Patient information:** the patient, a 45-year-old smoker with a 50-pack-year history and prior incarceration in 1998 and 2005, was hospitalized in October 2023 in the pulmonology department for asthenia, fever, night sweats, and exertional dyspnea evolving for several months. Upon questioning, there was a history of tuberculosis exposure from a neighbor.

**Timeline of current episode:** given the respiratory symptoms, a chest X-ray was performed, revealing bilateral infiltrates and pulmonary nodules. Sputum testing for *Mycobacterium tuberculosis* confirmed the diagnosis of pulmonary tuberculosis. Upon pre-therapeutic assessment, the patient had normochromic normocytic regenerative anemia at 11 g/dL, normal renal function, and normal liver function. After 20 days of anti-tuberculosis quadritherapy (isoniazid, rifampicin, pyrazinamide, and ethambutol), he developed respiratory discomfort with the onset of lower limb edema.

**Clinical findings:** on examination, he had a blood pressure of 140/90 mmHg, soft white pitting edema of the lower limbs, and on urine dipstick, proteinuria at 3 crosses and hematuria at 3 crosses were noted.

**Figure 1:**
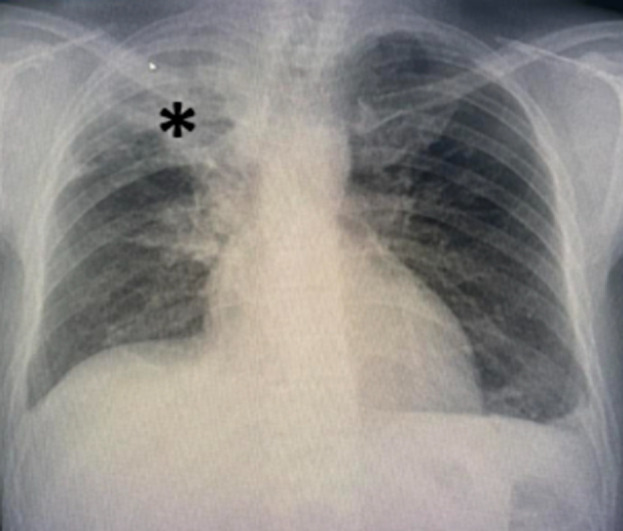
chest radiograph of the patient; right apical and supraclavicular heterogeneous opacities with interstitial and nodular infiltrates (*)

**Figure 2:**
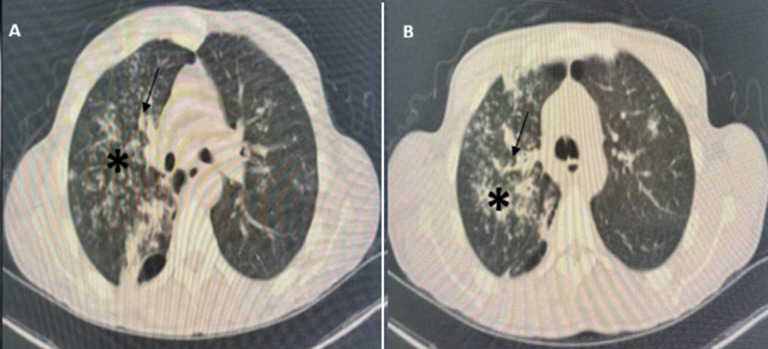
A,B) thoracic computed tomography (CT) of both lungs of the patient; consolidation with centrilobular branching micronodules, consistent with a tree-in-bud pattern, predominantly affecting the right upper lobe (*); peribronchovascular thickening (↓)

**Diagnostic assessment:** laboratory tests revealed acute renal failure with a creatinine level of 199 µmol/l and nephrotic syndrome with proteinuria at 11 g/24 hours, albuminemia at 19 g/dl, and proteinemia at 42 g/dl. Renal ultrasound showed two kidneys of normal size with good corticomedullary differentiation. Initially, rifampicin-induced nephrotoxicity was suspected, leading to its discontinuation. Renal function deteriorated rapidly, with levels increasing from 199 µmol/l to 504 µmol/l over 10 days, and hemoglobin decreased to 7.7 g/dl by December 16, 2023. An emergency chest X-ray revealed findings suggestive of pulmonary tuberculosis with signs of activity but no evidence of intra-alveolar hemorrhage (Figure 1). Thoracic computed tomography (CT) showed consolidation with centrilobular branching micronodules, consistent with a tree-in-bud pattern, predominantly affecting the right upper lobe, associated with peribronchovascular thickening (Figure 2). In case of acute nephritic syndrome, we performed autoimmune serologies (ANCA, ANA, anti-GBM antibodies, and complement), which were negative. Serology for hepatitis B and C was negative.

**Figure 3:**
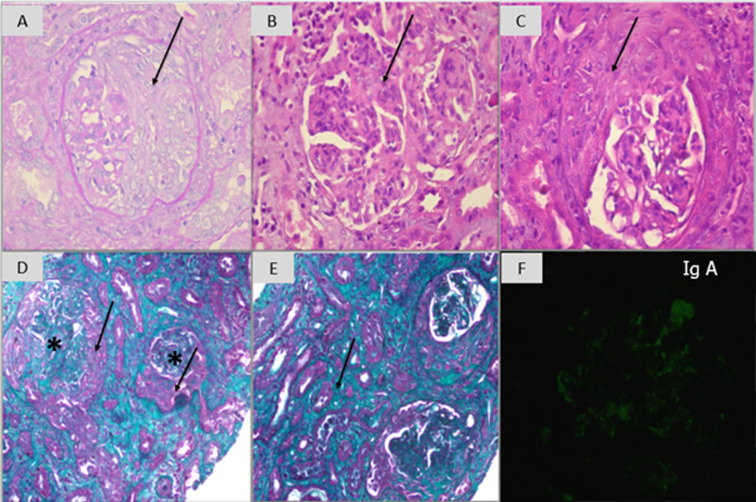
light microscopy findings from the patient´s renal biopsy; A) periodic acid shift X 200: segmental cellular crescent (↓); B) hematoxylin eosin X 400: mesangial hypercellularity (↓); C) hematoxylin eosin X 400: segmental cellular crescent (↓); D) Masson’s trichrome X 100: circumferential extracapillary crescent (↓), mesangial hypercellularity (*); E) Masson’s trichrome X 100: interstitial fibrosis and tubular atrophy (↓); F) immunofluorescence: mesangial deposits of IgA

**Diagnosis:** we completed a renal biopsy that yielded 2 fragments: one cortical and one cortico-medullary, allowing the examination of 14 glomeruli (Figure 3). All the glomeruli exhibited extracapillary proliferation, forming cellular or fibrocellular crescents. The basement membranes were thin but ruptured in some glomeruli, with the presence of fibrinoid necrosis. There was associated mesangial hypercellularity. The interstitium showed loose fibrosis with an inflammatory infiltrate. Immunofluorescence revealed mesangial deposits of IgA. This was identified as IgAN with diffuse crescents, classified as (M1E1S1T0C2) according to the Oxford classification.

**Therapeutic interventions:** the patient received three boluses of Solumedrol followed by oral corticosteroid therapy at a dose of 1 mg/kg/day, then a gradual decrease to 5 mg/kg. The antituberculous quadruple therapy was extended to 2.5 months and then followed by dual therapy. *Mycobacterium tuberculosis* testing was conducted every 15 days, yielding negative results.

**Follow-up and outcome of interventions:** the course was marked by a significant improvement in creatinine levels, reaching 262 µmol/L on March 29, 2024, but the nephrotic syndrome persisted.

**Patient perspective:** the patient expressed that his overall condition has improved, that he is no longer experiencing asthenia or respiratory discomfort, but he still has lower limb edema. He is also relieved that he is no longer dependent on dialysis.

**Informed consent:** the patient was informed about the publication of his case and assured that his anonymity would be strictly maintained. Written informed consent was obtained.

## Discussion

The pathophysiological mechanisms explaining the association between IgAN and tuberculosis have not been well elucidated [5,6]. The hypothesis is an activation of B lymphocytes with the production of hypoglycosylated IgA1 and/or antiglycan antibodies, leading to the formation of immune complexes that then deposit in the kidneys´ glomerular mesangium and elicit an inflammatory reaction with tissue damage [3-6]. Immunoglobulin A nephropathy has been associated with various tuberculous locations: pulmonary, as was the case with our patient, as well as peritoneal, osseous, lymphatic, cutaneous, and hepatic [5]. However, distinguishing primary IgAN coinciding with tuberculosis from tuberculosis-induced IgAN remains challenging. Our case highlights the need to perform a kidney biopsy in the presence of nephrological warning signs during a tuberculosis infection to rule out the presence of glomerulonephritis, including IgAN. In a 2016 literature review of 10 patients with IgA nephropathy associated with tuberculosis, 9 patients had focal or moderate mesangial proliferation on renal histology [5]. A 27-year-old patient had thickening of mesangial axes without mesangial proliferation [5]. None of these patients had extracapillary proliferation [5]. In 2019, a case of extracapillary IgA glomerulonephritis associated with pleural tuberculosis was reported. Renal biopsy showed crescent formation and fibrinoid necrosis in 12 out of 21 glomeruli [2]. The particularity of our patient was the severity of the histological involvement, with extracapillary proliferation extending to all the glomeruli. The antituberculous treatment, by reducing the viral load, will decrease the formation of immune complexes, explaining the clinical remission in the majority of reported cases of IgAN associated with tuberculosis under antituberculous treatment alone [6].

However, glucocorticoids can facilitate the spread of tuberculosis, yet their prescription is sometimes essential [6], as was the case with our patient given the significant diffuse extracapillary proliferation threatening renal prognosis. We considered prescribing cyclophosphamide for the patient, but due to the risk of infection exacerbation or dissemination, we decided not to. For our patient, the course was marked by recovery from tuberculosis, partial improvement in renal function, and persistence of nephrotic syndrome, indicating partial remission and possible irreversible glomerular damage. In a 2016 literature review of 10 patients with IgA nephropathy associated with tuberculosis, the course was marked by recovery from tuberculosis in all cases. From a nephrological perspective, a decrease in proteinuria was observed in all patients, though it did not become negative in 3 cases, hematuria disappeared in 9 patients, and chronic renal insufficiency persisted in 4 patients after an average follow-up duration of 15 months and 3 days. For the patient with extracapillary IgA glomerulonephritis, who most closely resembles our case, the course was marked by recovery from pleural tuberculosis but without recovery of kidney function, requiring continued hemodialysis [2]. The main strength of our case lies in the description of a rare association between tuberculosis and a severe form of IgA nephropathy with extracapillary proliferation. This coexistence represented a true diagnostic and therapeutic challenge, given the absence of clear recommendations in such a context. In addition, the renal biopsy was performed promptly, which contributed to an early and accurate diagnosis. However, the main limitation of our report is that the patient has been lost to follow-up since March 2024, which prevented assessment of the long-term clinical and renal outcomes.

## Conclusion

This case highlights the rare coexistence of pulmonary tuberculosis and crescentic IgAN, a combination that presents both diagnostic and therapeutic challenges. The main question that arises is whether this represents a primary IgAN coinciding with tuberculosis or a tuberculosis-induced IgAN. Managing active tuberculosis alongside severe glomerulonephritis requires a careful balance between infection control and immunosuppression. In the absence of specific guidelines, this case demonstrates that individualized corticosteroid therapy may lead to partial remission when carefully balanced with anti-tuberculosis treatment, despite severe initial histologic lesions.
